# Actively replicating West Nile virus is resistant to cytoplasmic delivery of siRNA

**DOI:** 10.1186/1743-422X-2-53

**Published:** 2005-06-28

**Authors:** Brian J Geiss, Theodore C Pierson, Michael S Diamond

**Affiliations:** 1 Departments of Medicine, Washington University School of Medicine, 660 South Euclid Avenue, Box 8051, St. Louis, MO 63110, USA; 2 Molecular Microbiology, Washington University School of Medicine, 660 South Euclid Avenue, Box 8051, St. Louis, MO 63110, USA; 3 Pathology & Immunology, Washington University School of Medicine, 660 South Euclid Avenue, Box 8051, St. Louis, MO 63110, USA; 4 Department of Microbiology, University of Pennsylvania, Philadelphia, PA, 19104, USA

## Abstract

**Background:**

West Nile virus is an emerging human pathogen for which specific antiviral therapy has not been developed. Recent studies have suggested that RNA interference (RNAi) has therapeutic potential as a sequence specific inhibitor of viral infection. Here, we examine the ability of exogenous small interfering RNAs (siRNAs) to block the replication of West Nile virus in human cells.

**Results:**

WNV replication and infection was greatly reduced when siRNA were introduced by cytoplasmic-targeted transfection prior to but not after the establishment of viral replication. WNV appeared to evade rather than actively block the RNAi machinery, as sequence-specific reduction in protein expression of a heterologous transgene was still observed in WNV-infected cells. However, sequence-specific decreases in WNV RNA were observed in cells undergoing active viral replication when siRNA was transfected by an alternate method, electroporation.

**Conclusion:**

Our results suggest that actively replicating WNV RNA may not be exposed to the cytoplasmic RNAi machinery. Thus, conventional lipid-based siRNA delivery systems may not be adequate for therapy against enveloped RNA viruses that replicate in specialized membrane compartments.

## Background

West Nile virus (WNV) is a significant human and veterinary mosquito-borne pathogen that has rapidly spread across North America. Humans develop a febrile illness and a small subset progress to meningitis or encephalitis syndromes [1]. Currently, no specific therapy or vaccine has been approved for treatment or prophylaxis of WNV infection in humans.

WNV is an enveloped virus with an 11-kilobase positive strand RNA genome. It is translated directly from the genomic RNA as a single polyprotein and cleaved by cellular and viral proteases into ten mature proteins, three structural (C, M, and E) and seven non-structural (NS1, NS2A, NS2B, NS3, NS4A, NS4B, and NS5) proteins [2,3]. Virus entry occurs by endocytosis after the E protein interacts with cellular receptor(s). Genomic viral RNA traffics to the endoplasmic reticulum (ER), where WNV protein translation and RNA replication occur [4]. The positive strand genomic WNV RNA that associates with the ER is competent for translation and transcription of negative strand RNA. WNV and related flaviviruses induce ER membrane proliferation and reorganization, and replicating viral RNA has been observed at these membranous structures [5-7]. Disruption of WNV protein translation and/or RNA replication blocks the viral lifecycle and aborts infection.

RNA interference (RNAi) is a cellular process that specifically degrades RNA within the cytoplasm of cells in a sequence-specific manner [8]. RNAi occurs in plants [9], nematodes [10], parasites [11,12], insects [13], and mammalian cells [14,15] and is believed to function as a regulator of cellular gene expression and possibly as an innate defense against RNA viruses [16]. RNAi uses double stranded RNA (dsRNA) to target and degrade sequence-specific single-stranded RNA. The cytoplasmic ribonuclease DICER recognizes and cleaves long dsRNA molecules into 21 to 30 base pair small interfering RNA (siRNA) molecules; these associate with the RNA Induced Silencing Complex (RISC) to target and degrade complementary single-stranded RNA molecules [8].

RNAi has been used as a method to transiently disrupt various gene products to study their function [14,15,17-20]. Many mammalian viruses appear susceptible to treatment with exogenous siRNA. Cells that express virus-specific siRNA are resistant to infection by WNV [21], poliovirus [22,23], influenza A [21,24], HIV [25] and hepatitis C [26,27]*in vitro*. Administration of siRNAs *in vivo *has modestly reduced hepatitis B antigen production [28,29] and influenza A virus infection [30,31]. The sequence specific activity of siRNA against viruses has led to great interest in its potential as a new class of antiviral therapy. Nonetheless, there may be limitations with this approach as *in vivo *RNAi has not been demonstrated as effective as post-exposure therapy.

Previously, we demonstrated that transgenic expression of a sequence-specific siRNA prior to infection could efficiently inhibit WNV replication [21]. However, for a WNV-specific siRNA to be effective as a post-exposure therapeutic, it would need to inhibit infection in cells that are actively replicating WNV RNA. In this study, we evaluated the efficacy of siRNA against WNV that has already initiated active replication. Although cytoplasm-directed transfection of cells with siRNA prior to infection efficiently blocked WNV infection, administration after infection had little efficacy. Unlike plant viruses that encode active suppressors of RNA interference [32-34], WNV did not appear to actively inhibit the RNAi response, but rather avoided degradation by replicating in a manner that was inaccessible to the RNAi machinery.

## Results

### In vitro generated siRNA inhibits WNV infection in cells

**Figure 1:**
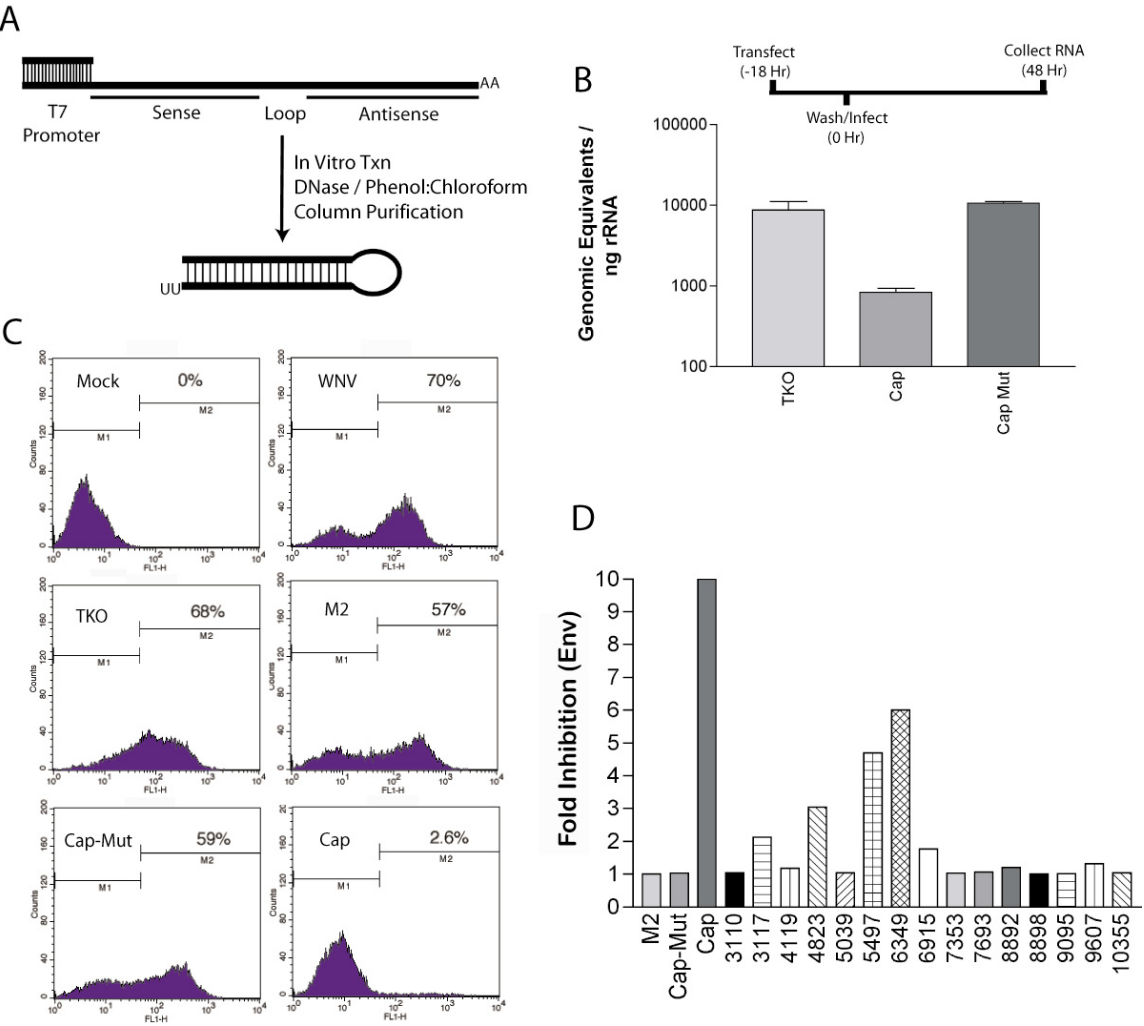
**WNV is susceptible to siRNA pretreatment. A. **Scheme for generation of small hairpin siRNAs. **B. **Cap siRNA specifically inhibits WNV RNA accumulation. Huh7.5 cells were mock transfected or transfected with Cap siRNA or Cap Mut siRNA. Eighteen hours later cells were infected with WNV at MOI 0.1. Forty-eight hours later cells were collected and total RNA was recovered. WNV RNA was measured by quantitative real time RT-PCR. The results are an average of three independent experiments and the error bars indicate standard error of the mean. **C. **Capsid siRNA specifically inhibits WNV E protein expression. Huh7.5 cells were mock transfected or transfected with M2 siRNA, Cap siRNA, or Cap Mut siRNA as described above. Forty-eight hours after infection, cells were collected and processed for flow cytometry using anti-WNV envelope protein antibody E1. The results are one representative example of three independent experiments. **D. **Inhibitory activity of different WNV-specific siRNA. Huh7.5 cells transfected with the indicated siRNA, infected with WNV, and then analyzed for viral antigen as described in Materials and Methods. The fold inhibition was calculated after dividing the percentage of antigen positive cells from mock-transfected cells by the percentage of antigen positive cells from siRNA transfected cells. The results are one representative example of two independent experiments.

We have previously demonstrated that plasmid expressed hairpin siRNA efficiently inhibited infection of WNV in mouse and human cell lines [21]. Because a therapeutic application of exogenously delivered rather than plasmid-expressed siRNA may be more clinically relevant, we assessed the inhibitory activity of *in vitro *transcribed hairpin siRNA against WNV infection. A 21-nucleotide region of the WNV capsid gene (nucleotides 312–332; Cap) was initially targeted, as this region is conserved among all WNV strains and lacks homology to known cellular genes. To demonstrate the specificity of Cap siRNA, a hairpin siRNA that targets the Influenza A M2 gene (nucleotides 18–38, M2) [21] and a mutated version of Cap siRNA (Cap Mut) that had 4 changes were also designed (Table 1). Our *in vitro *transcription strategy employed partially duplexed oligonucleotides containing a double stranded T7 promoter sequence (Fig 1A).

**Table 1:** Small interfering RNA

Name	Virus	Start Nucleotide	Target Sequence
Cap	WNV Lineage I	312	gaacaaacaaacagcgatgaa
Cap-Mut	WNV Lineage I	312	gaagaaagaaagaccgatgaa
M2	Influenza A M2	18	ggtcgaaacgcctatcagaaa
3110	WNV Lineage I	3110	gggcagttctgggtgaagt
3317	WNV Lineage I	3317	ctacggtcaccctgagtga
4119	WNV Lineage I	4119	gaggagcaagtctgctatgc
4823	WNV Lineage I	4823	gtgtcaaggaggatcgact
5039	WNV Lineage I	5039	gacggtgatgtgattgggct
5497	WNV Lineage I	5497	gcagcaagaggttacattt
6337	WNV Lineage II	6337	gttgaagtcatcacgaagt
6349	WNV Lineage I	6349	gtggaagtcatcacgaagc
6915	WNV Lineage I	6915	caacgagatgggttggcta
7353	WNV Lineage I	7353	gaagaacgctgtagtggat
7693	WNV Lineage I	7693	ggacgcaccttgggagaggt
8892	WNV Lineage I	8892	ggtcaacagcaatgcagct
8898	WNV Lineage I	8898	cagcaatgcagctttgggt
9095	WNV Lineage I	9095	gaagcagagccatttggtt
9607	WNV Lineage I	9607	gggaaaggacccaaagtca
10355	WNV Lineage I	10355	gagagatatgaagacacaac

siRNA were generated against 19–21 nucleotide sequences corresponding to the target region of different parts of the New York 1999 WNV genome. Sequences were chosen using the SciTools RNAi design program and compared against the GenBank database to exclude sequences that may affect cellular genes.

Human Huh7.5 cells were used because they were efficiently transfected with siRNA and infected with WNV. Huh-7.5 were transfected with Cap or Cap-Mut siRNA, infected with a New York strain of WNV at 18 hours after transfection, and analyzed 48 hours post-infection for levels of viral RNA by RT-PCR (Fig 1B). Pretreatment of Huh7.5 cells with Cap siRNA resulted in approximately 1 log reduction of WNV RNA, whereas pretreatment of Huh7.5 cells with Cap-Mut siRNA showed no significant reduction of WNV RNA. To confirm that RNAi also decreased WNV antigen production, siRNA-transfected Huh7.5 cells were examined for WNV envelope protein expression at 48 hours post infection. Approximately 70% of mock or TKO treated Huh-7.5 cells were positive for WNV antigen, levels comparable to that observed in cells transfected with either M2 (57% positive) or Cap-Mut (59% positive) siRNAs (Fig 1C). In contrast, less than 3% of Cap siRNA transfected cells stained positive for WNV E antigen. Thus, *in vitro *generated sequence-specific hairpin siRNA efficiently and specifically blocked WNV RNA and antigen production in mammalian cells.

To demonstrate that siRNAs targeting different regions of the WNV genome could inhibit infection, multiple siRNAs were designed spanning the nonstructural genes of WNV (Fig 1D). Two siRNAs (5497 and 6349) targeted to the nonstructural proteins reduced WNV envelope expression by at least 4-fold. Despite using an siRNA prediction algorithm, many of the siRNAs demonstrated little ability to inhibit envelope protein expression, possibly due to secondary structure in the WNV genomic RNA. Interestingly, treatment with combinations of siRNA did not show appreciably greater inhibition than treatment with either siRNA alone (data not shown).

### Timing of siRNA treatment affects effectiveness against WNV

**Figure 2:**
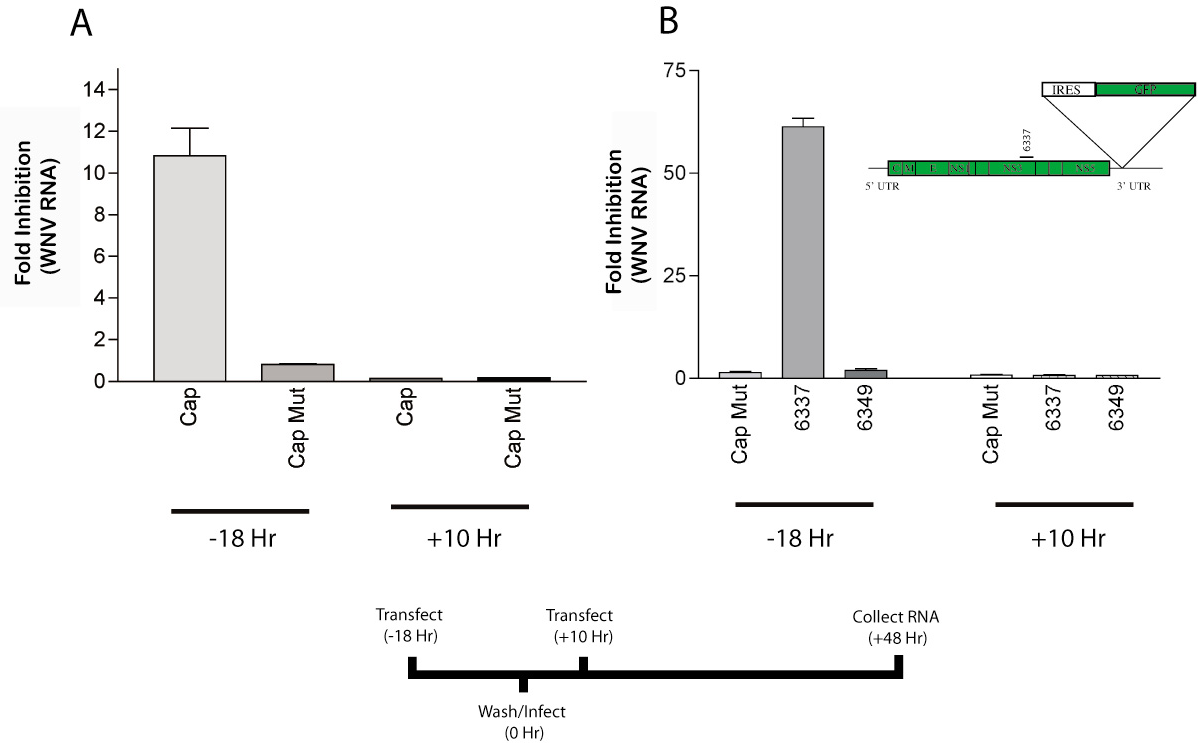
**WNV becomes resistant to RNAi after infection. A. **Huh7.5 cells were mock transfected or transfected with Cap or Cap Mut siRNA at the indicated times before or after WNV infection. Forty-eight hours after infection cells were harvested and WNV RNA levels were determined by quantitative real-time RT-PCR. The results are an average of three independent experiments and error bars indicate standard error of the mean. **B. **Induction of RNAi resistance by an attenuated lineage II WNV. *Inset*. Genomic structure of the attenuated lineage II WNV, which includes an IRES-controlled GFP insertion in the 3' UTR. 6337 denotes the target region of the lineage II specific siRNA. Attenuated WNV becomes resistant to siRNA after infection is established. Huh7.5 cells were transfected with Cap Mut, 6349, or 6337 siRNA at the indicated times prior to or after infection. Forty-eight hours after infection total RNA was collected and viral RNA was assessed as in Fig 1.

siRNA therapy in a clinical setting likely would require treatment after WNV infection has occurred. Because of this, we assessed the ability of siRNA to block WNV RNA before and after infection (Fig 2A). Huh7.5 cells were transfected with siRNAs 18 hours before infection or at 10 hours after infection. Cells were not transfected at very early times after infection because the TKO transfection reagent interfered with the ability of WNV virus to infect cells in a time-dependent manner (B. Geiss and M. Diamond, unpublished observation). By ~10 hours post-infection virus had entered cells and were no longer affected by the TKO reagent. Total RNA was harvested 48 hours post-infection and analyzed for genomic WNV RNA content. As expected, pretreatment of cells with Cap siRNA, but not Cap Mut siRNA, resulted in a 10-fold reduction in WNV RNA levels. Strikingly, addition of Cap siRNA 10 hours after infection resulted in no reduction of WNV RNA. The lack of inhibitory effect of RNAi at late times after infection was not due to the emergence of resistant mutants: sequence analysis of multiple viral isolates at 48 hours post-infection from cells that had been transfected with Cap or 6349 siRNA demonstrated no mutations in the targeted viral sequences (data not shown). Thus, WNV, in contrast to poliovirus [35], did not appear to mutate to evade siRNA-triggered degradation.

### The rate of viral replication does not affect RNAi resistance

The establishment of siRNA resistance could in part, be due to the ability of a rapidly replicating WNV to saturate the RNAi degradation machinery. To test if the replication rate affected siRNA resistance, we used an attenuated lineage II WNV that contains a GFP marker gene inserted into the 3' UTR and replicates more slowly than wild-type lineage I or II WNV [36]. Because the nucleotide sequence of the lineage II WNV was different than the lineage I WNV, a new sequence-specific siRNA was designed (6337) that targeted the analogous region on NS3 as siRNA 6349. Huh7.5 cells were transfected with Cap-Mut, 6349, or 6337 at 18 hours prior to or 10 hours after infection with the attenuated lineage II WNV, and viral RNA content was determined at 48 hours post-infection (Fig 2B). As expected, pretreatment with either Cap Mut or the lineage I-specific 6349 siRNA did not inhibit replication, whereas pretreatment with 6337 siRNA strongly blocked replication (~ 60-fold). In contrast, treatment with any of the three siRNAs 10 hours after infection demonstrated no inhibitory effect. Thus, a WNV strain with a lower replication rate similarly resisted the inhibitory effects of RNAi soon after replication was established.

### The mode of introduction of siRNA affects the ability to establish RNAi

Based on the timing experiments, the establishment of resistance to RNAi correlated with the onset of WNV RNA replication [37]. Shortly after infection, flaviviruses induce vesicular membrane proliferation that becomes the site of viral RNA replication [6,7,38]. Because the lipid-based transfection reagent targets nucleic acids to the cytoplasm (Mirus Corp, personal communication), the presence of additional membranes between the viral RNA and the cytoplasm could prevent the siRNA from reaching the actively replicating WNV RNA complex. Electroporation, in contrast, transiently opens pores in cellular membranes [39] allowing nucleic acids and cytoplasmic components to cross membranous structures such as the nucleus, endoplasmic reticulum, and potentially the membranous vesicles induced by WNV.

**Figure 3:**
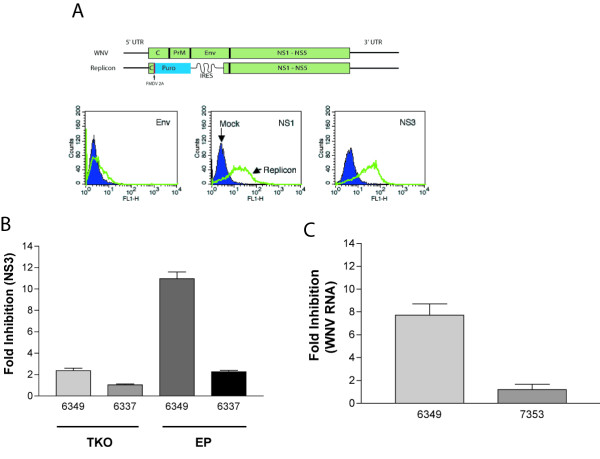
**Mode of siRNA introduction influences WNV RNAi susceptibility. A. **Huh7.5-Rep cells. (*Top*) Diagram of the genetic structure of the pWN5'Pur replicon. (*Bottom*) Flow cytometric analysis of Huh7.5 cells that express the pWN5'Pur replicon. Only non-structural proteins (e.g., NS1 and NS3 but not E) are expressed. **B. **siRNA treatment of Huh7.5-Rep cells. Huh7.5-Rep cells were mock-transfected, transfected with TKO reagent complexed with 6337 or 6349 siRNAs, or electroporated with 6337 or 6349 siRNAs. Three days later, cells were processed for viral NS3 protein expression by flow cytometry using anti-NS3 antibody (right). Fold inhibition of NS3 antigen production was determined using the formula (% NS3 positive mock electroporated / % NS3 positive siRNA electroporated). **C. **RNA analysis of Huh7.5-Rep cells electroporated with siRNA. Huh7.5-Rep cells were electroporated with 6349 or 7353 siRNA as described in Materials and Methods. Three days later, total cellular RNA was collected and viral RNA was assessed. Fold inhibition was determined by dividing the amount of viral RNA in mock electroporated samples to the amount of viral RNA in siRNA electroporated samples. The results are an average of three independent experiments and error bars indicate standard error of the mean.

To determine if the mode of delivery of siRNA affected RNAi resistance, we tested whether siRNA could inhibit replication of a persistently replicating subgenomic lineage I WNV replicon; this cell line (Huh7.5-Rep) expresses the non-structural but lacks the structural proteins of WNV (Fig 3A). Huh7.5-Rep cells were transfected with 6337 and 6349 siRNAs using a lipid-based reagent or by electroporation, cultured, and assayed for reduction of NS3 antigen expression 72 hours later (Fig 3B). Replicon-expressing cells that were transfected with siRNA using the lipid-based reagent showed no significant reduction in viral protein or RNA. In contrast, using electroporation, NS3 protein levels were reduced by approximately 10-fold by 6349 siRNA, but not by the lineage II-specific 6337 siRNA. The reduction of NS3 antigen levels correlated with ~7.5-fold decreases in replicon RNA levels in the presence of 6349 (Fig 3C).

### Cellular localization of siRNA

**Figure 4:**
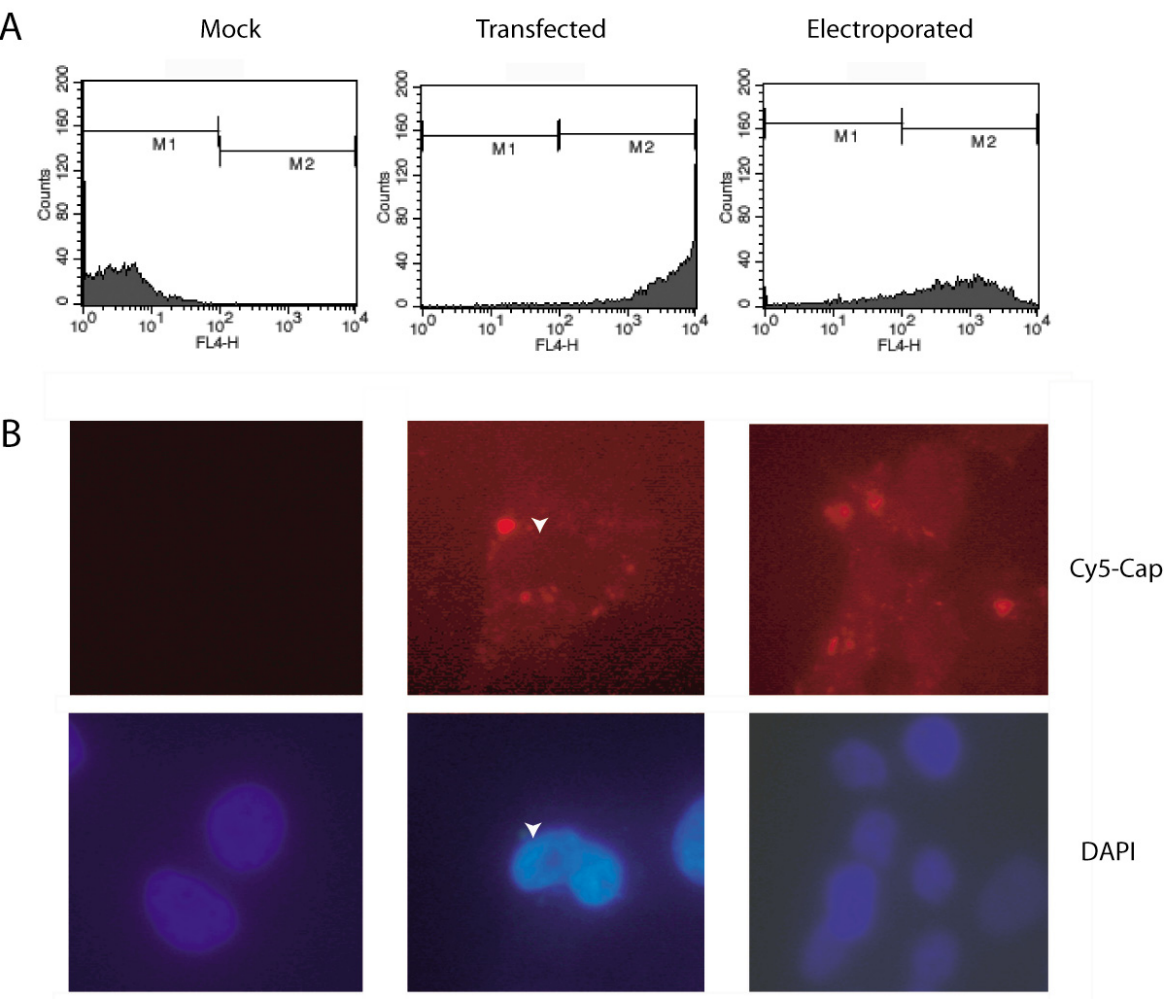
**Localization of siRNA. **Huh7.5 cells were transfected with Cy5 labeled Cap siRNA using the lipid TKO reagent (*middle panels*) or by electroporation (*right panels*). Eighteen hours later, cells were collected and analyzed for Cy5 fluorescence by (**A**) flow cytometry or (**B**) fluorescence microscopy as described in Materials and Methods. Arrows denote the position of the nucleus. The gain in the Cy5 micrograph of electroporated cells was increased to compensate for lower levels of intracellular siRNA as compared to lipid-transfected samples.

The preceding data suggested that lipid-mediated transfection delivered siRNA into the cytoplasm, whereas electroporation at least transiently exposed replicating viral RNA to the RNAi response. However, it remained unclear whether the amount of siRNA delivered or the localization determined its ability to inhibit WNV RNA. To assess the relative amount and distribution of siRNA after transfection or electroporation, Huh7.5 cells were transfected or electroporated with Cy5-labeled Cap siRNA and analyzed 18 hours later for Cy5 expression by flow cytometry and localization by fluorescence microscopy (Fig 4). Although all transfected cells were positive for Cy5 fluorescence, the signal was significantly higher in lipid-transfected cells than in electroporated cells (Fig 4A, geometric mean fluorescence intensity 4370 compared to 766). Microscopic analysis of lipid-transfected cells showed Cy5 signal primarily in the cytoplasm, with exclusion of Cy5 from the nucleus (Fig 4B, middle panels). In contrast, Cy5 signal was observed diffusely throughout the cell after electroporation (Fig 4B, right panels), suggesting that electroporation effectively delivered siRNA across intracellular membranes. Taken together, our data suggests that the cellular localization of siRNA appears more important than the absolute amount of siRNA delivered into the cell in determining its effectiveness against actively replicating WNV RNA.

### RNAi against other mRNAs is intact in WNV-infected cells

**Figure 5:**
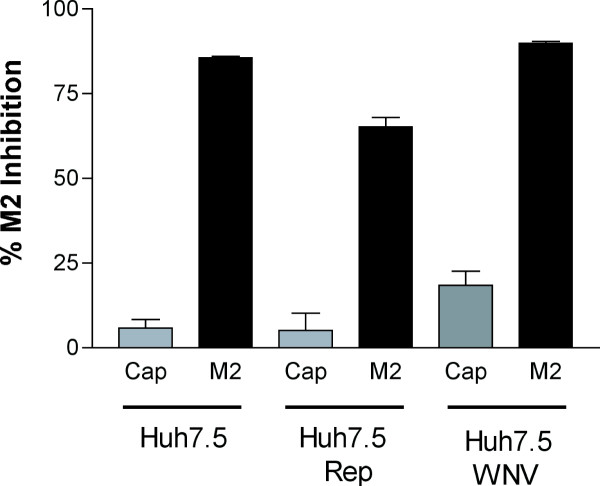
**RNAi is active in WNV infected cells**. RNAi of influenza M2 gene in cells that replicate WNV RNA. Huh7.5, Huh7.5-Rep, and WNV infected Huh7.5 cells (8 hours post infection) were transfected with pCM2 and Cap or M2 siRNA as described in Materials and Methods. 24 hours later cells were processed by flow cytometry for M2 expression using antibody 14C2. The percentage of M2 inhibition was calculated according to the following formula: (1 – (% M2 expression of siRNA-transfected cells / % M2 expression in cells transfected with transfection vehicle only) × 100). The results are an average of three independent experiments and error bars indicate standard error of the mean.

Although the mode and location of siRNA introduction could affect the sensitivity of actively replicating WNV to RNAi, we speculated that WNV could additionally evade RNAi by directly inhibiting one or more steps of the RNAi pathway. Targeted inhibition of the RNAi pathway has been observed in plant viruses, and has been recently reported with several mammalian viruses including LaCrosse virus, adenovirus, and influenza A virus [32-34,40-43]. To determine if WNV replication directly attenuated the RNAi response, we tested the efficiency of siRNA-mediated inhibition of Influenza A virus M2 protein expression in cells that actively replicated WNV genomes (Fig 5). Mock-infected Huh7.5 cells, WNV-infected Huh7.5 cells (10 hours post-infection), and Huh7.5-Rep cells were co-transfected with an M2 expression plasmid and either Cap or M2 siRNA. Twenty-four hours later, cells were analyzed for M2 expression by flow cytometry. As expected, transfection of Cap siRNA did not significantly affect the expression of M2 in Huh7.5, Huh7.5-Rep, or WNV infected Huh7.5 cells. However, transfection of M2 siRNA effectively reduced the expression of the M2 protein in all cell types, including those that actively replicated WNV RNA. Thus, WNV replication *per se *did not affect the establishment of RNAi of a heterologous gene.

### Viral translation is necessary for WNV RNAi resistance

**Figure 6:**
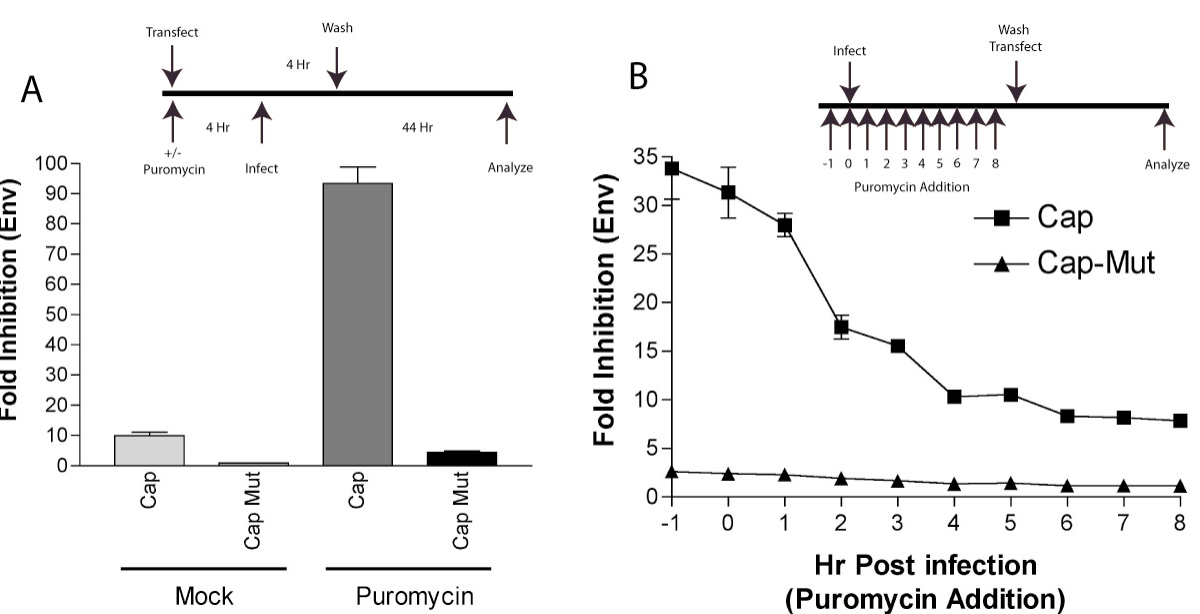
**WNV RNAi resistance is dependent of viral translation early in infection. A. **Puromycin does not interfere with RNAi. Huh7.5 cells were mock-treated or treated with 6 μg/ml puromycin and transfected with Cap or Cap Mut siRNA for 4 hours. Cells were washed twice, the puromycin replaced, and cells were infected with WNV at MOI 0.1. Four hours later cells were washed twice and replaced with medium that lacked puromycin. Forty-eight hours after infection, cells were collected and analyzed for WNV envelope protein expression by flow cytometry. Fold inhibition was calculated as described above. The results are an average of three independent experiments and error bars indicate standard error of the mean. **B. **Puromycin time course. Huh7.5 cells were infected with WNV (MOI = 0.01), and puromycin was added at the indicated times before or after infection. At 9 hours post-infection cells were washed and transfected with Cap or Cap Mut siRNA. Forty-eight hours later, WNV envelope protein expression was assessed by flow cytometry. Fold inhibition was calculated as described in Fig 3B. The results are an average of three independent experiments and error bars indicate standard error of the mean.

The experiments above suggest that resistance to RNAi by WNV occurs in the setting of ongoing viral replication. During the *de novo *infection of a cell, translation of the input positive viral RNA strand is required before replication occurs [2]. To more finely dissect the kinetics of RNAi resistance with respect to the initiation of RNA replication, we added puromycin, a reversible inhibitor of protein chain elongation. Because puromycin inhibits cellular and viral protein translation, we first assessed how it independently affected the establishment of RNAi. Cap or Cap Mut siRNA were transfected into cells in the presence of puromycin. Four hours later, cells were infected with WNV for an additional four hours, and then free virus and puromycin were removed by serial washing of infected monolayers. Forty-eight hours after initial infection, cells were analyzed for WNV envelope protein expression (Fig 6A). When translation of full-length viral protein was reduced by puromycin, Cap siRNA more effectively inhibited WNV antigen production (90-fold versus 10-fold reduction), suggesting that cellular translation was not necessary for priming the RNAi response and that delay of onset of WNV translation enhanced the efficiency of siRNA-mediated inhibition. Importantly, the inhibition was sequence-specific as no significant decrease in viral antigen expression was observed with the Cap Mut siRNA.

To define the kinetics of RNAi resistance around the time of initial viral replication, a puromycin time course was performed (Fig 6B). Cells were infected with WNV at MOI 0.01 and puromycin was added to Huh7.5 cells at different times (-1, 0, 1, 2, 3, 4, 5, 6, 7, or 8 hours) before or after infection. At 9 hours after WNV infection, all cells were transfected with Cap or Cap Mut siRNA and puromycin was removed from the medium. Cells treated with puromycin from -1 to 1 hours post-infection were greatly protected against WNV infection. However, significant attenuation of RNAi emerged when puromycin was added just four hours after infection. These results suggest that the induction of RNAi resistance by WNV depends on translation of viral polyprotein and occurs as early as 4 hours after infection.

## Discussion

In this paper we examine the ability of exogenous siRNA to inhibit the replication of WNV. Developing strategies for specific inhibition of WNV is an important goal as no current therapy exists for infected individuals. siRNA has been proposed as a potential therapy against several viruses, and we have previously demonstrated that plasmid based RNAi is effective against WNV *in vitro *[21]. Here, we tested the ability of exogenously generated siRNA to inhibit WNV infection, as this reagent may be more practical for clinical use because there is little possibility of adverse integration into a patient's genome. Using a conventional lipid-based delivery system that targets siRNA to the cytoplasm, we confirmed that pretreatment of cells prevented infection. However, resistance to RNAi was observed when siRNA was delivered after viral translation and replication had commenced. In contrast, when siRNA was delivered by electroporation, a technique that allows macromolecules to pass across intracellular membranes, it reduced viral replication in a sequence-specific manner even if active replication was already underway. The data in this manuscript provide a first description of flavivirus resistance to RNAi during infection, and suggests a possible mechanism: WNV resists exogenously-introduced siRNA by replicating in a compartment that is sequestered behind cellular membranes.

Poliovirus is a positive strand RNA virus that replicates its genome in the cytoplasm of infected cells [44] and although susceptible to siRNA treatment, may relieve the selective pressure from siRNA by accumulating mutations in the targeted region [22,23,35]. In contrast, despite sequencing multiple independent isolates, we were unable to identify any mutations in siRNA-targeted regions in WNV-infected or replicon-expressing cells that were exposed to inhibitory siRNA. Also with poliovirus, some of the RNAi resistance could be overcome by administration of multiple inhibitory siRNA to disparate regions of the genome [35]. However, this was not observed with WNV, as simultaneous delivery of multiple inhibitory siRNA did not affect the resistance to RNAi in WNV-infected or replicon expressing cells. Thus, unlike poliovirus, WNV does not appear evade RNAi by mutating its target sequences.

WNV polyprotein translation and RNA replication within hours of infection [45]. Treatment of cells with the protein chain elongation inhibitor puromycin confirmed that establishment of RNAi resistance depended on translation of the infectious viral RNA, and that this occurred within the first four hours of infection. Electroporation of siRNA into cells expressing actively replicating WNV replicons aborted replication, suggesting both a mechanism and a means to overcome WNV-induced RNAi resistance. Nonetheless, it is possible that the method of delivery independently affects the ability of the siRNA to prime the RNAi response. The route of delivery differs between TKO transfection and electroporation (endosome versus direct transfer across membranes), and a proportion of TKO transfected siRNA may remain in endosomes for extended periods of time after transfection. However, even though five-fold more siRNA was detected in cells transfected by the TKO method, no inhibition was observed in cells that had ongoing replication of WNV RNA. We favor an alternative explanation in which WNV replication complexes are physically sequestered in a *de novo *generated specialized membranous compartment that is inaccessible to the cytoplasmic RNAi machinery. However, if siRNA gains access to these compartments (e.g., by electroporation) the RNAi machinery can be primed for sequence-specific degradation of viral RNA. Consistent with this, several studies have indicated that the reorganization and proliferation of endoplasmic reticulum membranes induced by flaviviruses is essential for efficient replication [6,7,38]. Uchil and Satchidanandam [46] proposed a model of flavivirus RNA replication in which viral dsRNA is enclosed within a double membrane structure; such a model could explain our findings. When siRNA is introduced by transfection prior to WNV infection, the cytoplasmic RNAi machinery becomes primed, and efficiently degrades infectious viral RNA after nucleocapsid penetration but before translation. In contrast, when siRNA is introduced by lipid-based transfection several hours after infection, replicating viral RNA is sequestered from the cytoplasm where the RNAi response is primed, allowing near-normal levels of replication to occur. During electroporation, however, siRNAs may be delivered across membranes and into the lumen of the viral replication compartment. How the Dicer and RISC components gain access into the replication compartment remains unknown. Although some cytoplasmic proteins may translocate across membranes during electroporation, the large size of the RNAi machinery may limit transport across membrane structures. We speculate that a small amount of Dicer and RISC gains access to the lumen of the replication compartment during its formation, and become activated when siRNA are delivered via electroporation. Clearly, additional experiments are necessary to confirm the precise mechanism.

Because treatment with siRNA *in vivo *would occur after an infection has been established, post-infection administration of siRNA in cell culture may reasonably predict the therapeutic utility of siRNA against individual viruses. Although many recent reports, including our own [21-27], have documented that pretreatment of cells with siRNA effectively aborts infection, few studies have examined the effects of siRNA treatment on established virus infection *in vitro *or *in vivo*. For example, siRNA administration into mice 5 hours after Influenza A infection only modestly reduced viral titers [31]. Several groups have recently demonstrated that electroporation of hepatitis C (HCV)-specific siRNA reduced HCV RNA replication in cells expressing subgenomic replicon [26,27,47-50], results that are consistent with ours. In contrast, one study reported that siRNA transfection with oligofectamine, a lipid-based reagent, modestly reduced HCV protein expression and RNA replication in HCV-replicon expressing cells [50]. The disparity among results with lipid-based transfection systems may be reagent-based, as oligofectamine is reported to deliver a fraction of the siRNA across membranes (Invitrogen, personal communication) and thus, may transport small amounts of siRNA into the HCV replication compartment.

## Conclusion

The data presented here suggests that actively replicating WNV avoids the RNAi response by replicating in a manner that is inaccessible to cytoplasm-targeted delivery of siRNA. Consistent with this, we observed little therapeutic effect of siRNA against WNV *in vivo *in mice (B. Geiss, M. Diamond, unpublished observation). No protection against WNV was observed when mice were treated with siRNA 24 hours after infection [51]. This lack of siRNA-mediated therapeutic effect *in vivo *correlates with the induction of siRNA resistance that we observe *in vitro*. Future studies will address the role of flavivirus-induced membrane reorganization in RNAi resistance, and determine whether this mechanism is a common feature of other positive strand enveloped RNA viruses. Such information may inform the development of alternate delivery systems that allow siRNA to efficiently cross intracellular membranes and inhibit actively replicating enveloped viruses.

## Materials and methods

### Cells, viruses, and plasmids

Baby hamster kidney cells (BHK21-15 [52]) and human Huh-7.5 hepatoma cells (gift from C. Rice, New York, NY [53]) were cultured in Dulbecco's Modified Eagle Medium with 10% fetal bovine serum as previously described [52]. The lineage I (3000.0259, New York 2000) and the lineage II WNV strains have been described previously [54-56].

The lineage I WNV subgenomic replicon plasmid pWN5'Pur was generated from a genomic clone of the New York 1999 strain (plasmids pWN-AB1 and pWN-CG) provided by R. Kinney (Centers for Disease Control, Fort Collins, CO). pWN5'Pur was generated by deleting WNV nucleotides 181–2379 and fusing the first 31 amino acids of the capsid protein followed by the FMDV 2A autocleavage peptide [57] and the puromycin N-acetyl transferase (PAC) gene [58]. The EMCV IRES [53] was placed downstream of the PAC stop codon, so that translation of the WNV structural proteins begins at nucleotide 2380 (Methionine 794). The lineage II WNV genomic clone containing a Not I restriction site or an IRES-driven GFP have been described [36,56].

DNA template for replicon RNA transcription was prepared by linearization of pWN5'Pur with Xba I restriction endonuclease followed by phenol:chloroform extraction and ethanol precipitation. Replicon RNA was generated using the Amplicap T7 High Yield Message Maker kit (Epicenter Technologies, Madison WI). T7 RNA transcripts were electroporated into Huh7.5 cells as described below to generate Huh7.5-Rep cells. Huh7.5-Rep cells were stably selected with 5 μg/ml puromycin (Sigma-Aldrich, St. Louis MO). Reverse transcriptase PCR was performed as previously described [59,60] using primers 3026F (5'TGACTCGAAGATCATTGGAA) and 4496R (5'ATCCATATCTTCCAAGGTGC). Plasmid pCAGGS M2 was described previously [21].

### siRNA production, RNA and DNA transfection

siRNA were generated *in vitro *by run-off transcription from a partially double-stranded oligonucleotide template. Oligonucleotides that contained the T7 RNA polymerase promoter (5'AAATTTAATACGACTCACTATA) were annealed to a 75-mer oligonucleotide, which contained an antisense T7 RNA polymerase promoter sequence, 19–21 nucleotides corresponding to the target sequence (Table 1), a 10 nucleotide loop region, 19–21 nucleotides complementary to the target sequence, and two adenine residues (5'AA (sense 21) AACCAGAAGA (antisense 21) TATAGTGAGTCGTATTAAATTT). Targeted sequences were chosen using the SciTools RNAi design program (Integrated DNA Technology, Coralville, IA) and compared against the GenBank database to exclude sequences that may affect cellular genes. Polyacrylamide gel electrophoresis (PAGE)-purified oligonucleotides were purchased from Integrated DNA Technologies (Coralville, IA).

siRNA were transcribed using the MegaShortScript T7 Transcription Kit (Ambion, Austin, TX) according to the manufacturers recommendations with the exception that 200 additional units of T7 RNA Polymerase (Ambion) were included in each 20 μl reaction. RNA transcription reactions were carried out at 37°C for 1.5 hours, treated with DNAse I for 15 min, extracted with phenol and chloroform, desalted over ChromaSpin TE-10 columns (BD Biosciences, Palo Alto, CA), and stored at -80°C. siRNA were quantified by PAGE gel electrophoresis and UV spectroscopy. Cy5 labeled siRNA was generated by adding 1 mM Cy5-UTP (Amersham Biosciences, Piscataway, NJ) to transcription reactions. Huh7.5 cells were transfected at various times before or after infection with 1 μg siRNA using 5 μl Trans-IT TKO reagent (Mirus Corp., Madison, WI) according to the manufacturer's instructions. Electroporations were performed using a BTX ElectroSquarePorator as described [53]. In all electroporation experiments, 5 × 10^6 ^cells were electroporated in the presence of 50 μg siRNA. TKO transfected cells were washed twice with fresh DMEM media before infection with WNV. Viral antigen expression in WNV-infected cells was analyzed by flow cytometry (FACSCalibur, Becton-Dickinson) using the anti-WNV envelope E1 monoclonal antibody [61]. Monoclonal antibodies against NS1, (9NS1; K. Chung and M. Diamond, unpublished data) and NS3, (clone E1E6; R. Beatty and E. Harris, unpublished data) were used to detect viral antigen in cells that expressed WNV replicons. Goat-anti-mouse IgG -FITC (Sigma-Aldrich, St. Louis MO) was used to detect primary antibodies. Plasmid DNA transfections were performed using Trans-IT LT1 reagent (Mirus Corp., Madison WI) at a ratio of 8 μl transfection reagent / 1 μg plasmid DNA according to the manufacturers recommendation. For experiments involving co-transfection of plasmid and siRNA, plasmid DNA and siRNA were separately complexed with the appropriate transfection reagent, mixed together and incubated at room temperature for 15 minutes, and added to cells.

### Quantitative real-time reverse transcriptase PCR

WNV infected samples were collected at 48 hours post-infection, and total RNA was isolated using RNEasy RNA extraction columns (Qiagen, Valencia, CA) according to the manufacturer's instructions. Real-time reverse transcriptase PCR and quantitation of WNV transcripts was performed as previously described [62]. Quantitation of Lineage I replicon RNA was performed using a primer-probe set directed towards the 3' UTR WNV genome (forward primer 5'AGAGTGCAGTCTGCGATAGTGC; probe 5' Fam ACAAAGGCAAACCAACGCCCCA TAMRA; reverse primer 5'CCTTTCGCCCTGGTTAACA). Quantitation of Lineage II genome was performed using a primer set directed towards the 3' UTR of the Lineage II WNV genome (forward primer 5'AGAGTGCAGTCTGCGATAGTGC; probe 5' FAM ACAAAGGCAAAACATCGCCCCA TAMRA; reverse primer 5'CCCTTCTCCCTGGTTAACA).

### Fluorescence microscopy

Cy5-Cap transfected or electroporated Huh7.5 cells were plated onto Lab-Tek glass slides (Nalge Nunc, Naperville IL) and incubated for 18 hours at 37°C. Cells were fixed in cold 4% paraformaldehyde, washed extensively, permeabilized with 0.5% Triton X-100, and mounted in Prolong Gold Plus DAPI mounting reagent (Molecular Probes, Eugene OR). Slides were visualized and digitally captured using a Zeiss Axioskop microscope (Zeiss Microimaging, Thornwood, NY).

## Competing interests

The author(s) declare that they have no competing interests.

## Authors' contributions

BJG designed and constructed the subgenomic WNV replicons, designed and performed all experiments, and helped draft the manuscript. TCP designed, constructed, and tested the WNV-GFP clone and critically reviewed the manuscript. MSD and BJG designed the study, and MSD helped draft and critically review the manuscript.
